# Selections

**Published:** 1897-10

**Authors:** 


### SELECTIONS.

Alexander Galbraith, in a recent issue of the *Breeders' Gazette,* referred as follows to the different breeds of ponies:

The established breeds of ponies in the British Isles include the Shetland, Iceland, Exmoor, Welsh, New Forest, and West Highland, all of which may, practically speaking, be called pure-bred. The Shetland is the smallest pony, ranging generally from 36 to 44 inches, and is, on account of his low stature and docile disposition, by far the best children's pony. His characteristics are strength, hardiness, docility, and, when well bred, wonderfully good action. Provided by nature with the means of resisting the storms to which he is exposed in winter in the Shetland Isles, he is at that season as "shaggy as a sheep," but when warmer weather comes, and he gets into a better condition of flesh, he sheds off like all other horses. The prevailing colors are blacks and browns, with a sprinkling of bays and sorrels, and an occasional dun, piebald or skewbald.

The Iceland pony stands from 10 to 12 hands high, and is frequently sold as a Shetlander, but is by no means equal either in conformation or disposition. They are strongly built ponies, their heads being decidedly larger. Many are natural pacers, and quite a few of them are possessed of a rather stubborn disposition. Iceland ponies are much more numerous and much less valuable than Shetlands. They are all colors, including many skewbalds, or bay and white.

The Exmoor is a south of England pony, bred chiefly on the moorlands of Devonshire. They range from 11½ to 13 hands, the average being about 12 hands. They are of finer quality and more intelligent than the Iceland pony—in fact, superior every way, but being somewhat high-spirited are not as safe for children's use as the "wee Shetland shelty."

The Welsh pony is very similar to the Exmoor, but a trifle larger, say about 13 hands on an average. Bays and browns predominate in both breeds. The Welsh pony is full of mettle, and many of them have splendid action. The cross between a selected Welsh pony mare and a good 14 to 15-hands hackney stallion often produces the ideal cob-pony, which is the most useful animal on an English farm.

There are about three thousand breeding ponies in Hampshire,

England, of what is known as the New Forest breed. They range from $11\frac{1}{2}$ to $13\frac{1}{2}$ hands high, and are somewhat similar in many respects to the Welsh and Exmoor ponies, but have been repeatedly crossed with Arab blood during the last forty years. Both Queen Victoria and her late husband, Prince Albert, put Arab stallions into the New Forest at different times, and many excellent polo-ponies have been produced there, although Lord Arthur Cecil—a very good authority—is of the opinion that the larger of the Welsh ponies are equally good even for polo.

The last and largest of the pony breeds is the West Highland, which is indigenous to the west and northwest highlands of Scotland. They range generally from 13 to 14 hands high, are very stout and symmetrically built, resembling miniature draught-horses, which in point of fact they really are. Their disposition is like that of the Highlander himself—rather peculiar. For their height, they are immensely strong, very sound in wind and limb, mostly dark, solid colors, and they prefer walking to trotting at all times.

Of all pony-breeders in Great Britain, Lord Londonderry stands easily at the head for Shetlands. He has a large and carefully selected stud on the Island of Bressay, and has done much to improve the breed, and especially their action, during the last twenty-five years. At the recent Royal Show all the prize-winners, both male and female, in the Shetland section were bred by his lordship.

Mr. Christopher Wilson, of Rogmaden Park, Westmoreland, has the distincton of having created the modern show-pony by judicious selections and successful mating. His well-known stallion pony, Sir George, a miniature hackney, won first prize eight successive years at the Royal Show. He was crossed with his own daughters, granddaughters, and great granddaughters, with the most marvellous success—in fact, no ponies in England are comparable to what have been produced in this seemingly unnatural way. They have good constitution, plenty of bone of excellent quality, and most charming all-around action. When standing they resemble very small hunters; in action they are hackneys.

As proof of how such ponies are valued in England, I may state that in 1895 six mares, from yearlings upward, were sold under the hammer for the phenomenally high average of £721, about $3500 each. Who would not like to raise and sell ponies at such figures?

I had almost omitted to make mention of the Irish Connemara pony —a useful animal of from 12 to 14 hands high—that is said to have been descended from Spanish stock left in the country when the Armada was defeated by Great Britain.

